# Differential Specificity of Endocrine FGF19 and FGF21 to FGFR1 and FGFR4 in Complex with KLB

**DOI:** 10.1371/journal.pone.0033870

**Published:** 2012-03-19

**Authors:** Chaofeng Yang, Chengliu Jin, Xiaokun Li, Fen Wang, Wallace L. McKeehan, Yongde Luo

**Affiliations:** 1 Center for Cancer and Stem Cell Biology, Institute of Biosciences and Technology, Texas A&M Health Science Center, Houston, Texas, United States of America; 2 IBT Proteomics and Nanotechnology Laboratory, Institute of Biosciences and Technology, Texas A&M Health Science Center, Houston, Texas, United States of America; 3 School of Pharmacy, Wenzhou Medical College, Wenzhou, China; Ecole Normale Supérieure de Lyon, France

## Abstract

**Background:**

Recent studies suggest that betaKlotho (KLB) and endocrine FGF19 and FGF21 redirect FGFR signaling to regulation of metabolic homeostasis and suppression of obesity and diabetes. However, the identity of the predominant metabolic tissue in which a major FGFR-KLB resides that critically mediates the differential actions and metabolism effects of FGF19 and FGF21 remain unclear.

**Methodology/Principal Findings:**

We determined the receptor and tissue specificity of FGF21 in comparison to FGF19 by using direct, sensitive and quantitative binding kinetics, and downstream signal transduction and expression of early response gene upon administration of FGF19 and FGF21 in mice. We found that FGF21 binds FGFR1 with much higher affinity than FGFR4 in presence of KLB; while FGF19 binds both FGFR1 and FGFR4 in presence of KLB with comparable affinity. The interaction of FGF21 with FGFR4-KLB is very weak even at high concentration and could be negligible at physiological concentration. Both FGF19 and FGF21 but not FGF1 exhibit binding affinity to KLB. The binding of FGF1 is dependent on where FGFRs are present. Both FGF19 and FGF21 are unable to displace the FGF1 binding, and conversely FGF1 cannot displace FGF19 and FGF21 binding. These results indicate that KLB is an indispensable mediator for the binding of FGF19 and FGF21 to FGFRs that is not required for FGF1. Although FGF19 can predominantly activate the responses of the liver and to a less extent the adipose tissue, FGF21 can do so significantly only in the adipose tissue and adipocytes. Among several metabolic and endocrine tissues, the response of adipose tissue to FGF21 is predominant, and can be blunted by the ablation of KLB or FGFR1.

**Conclusions:**

Our results indicate that unlike FGF19, FGF21 is unable to bind FGFR4-KLB complex with affinity comparable to FGFR1-KLB, and therefore, at physiological concentration less likely to directly and significantly target the liver where FGFR4-KLB predominantly resides. However, both FGF21 and FGF19 have the potential to activate responses of primarily the adipose tissue where FGFR1-KLB resides.

## Introduction

The diverse FGF homologues constitute a large family of 22 distinct proteins in human that sense environmental cues to regulate cellular and metabolic homeostasis [1], [2], [3], [4], [5]. Their transmembrane receptor tyrosine kinase FGFR family encoded by 4 genes contains a plethora of isoforms through combinatorial alternative splicing of their gene products [5], [6], among which are the principal IIIb and IIIc isoforms for FGFR1–3 but not FGFR4. With heparan sulfate glycan motifs as the cofactor, the canonic FGFs including FGF1–10, 16–18, 20 and 22 are predominant for cell growth, survival, differentiation and migration that when aberrant may drive cancer progression [5], [7]. With the assistance from transmembrane cofactors Klotho (KL) and betaKlotho (KLB), the endocrine FGF19 subfamily including FGF19, 21 and 23 play important roles in control of bile acid synthesis and systemic lipid, glucose, energy and minerals metabolic homeostasis [8], [9], [10], [11].

Unlike the canonic FGFs that possess high affinity for matrix heparan sulfate (HS) motifs [5], [7], [12], [13], [14], [15] and thus act only locally as autocrine and paracrine factors, the FGF19 subfamily has extremely low affinity for HS, which permits their circulation as endocrine hormones to distal tissues/organs where the FGFR and KL or KLB are co-expressed [16], [17]. KL and KLB are single transmembrane domain proteins containing tandem glycosidase domains of unknown functions in the extracellular region and short intracellular tails [18], [19], [20]. KLB is expressed in liver, adipose tissue, pancreas and muscle, and KL in kidney and intestine [18], [21], [22], [23]. It has been thought that the endocrine effects of these hormonal FGFs are determined by expression patterns in adult tissues of themselves, FGFRs and cofactor KL/KLB, and by their specific interactions with different combinations of FGFRs and KL/KLB that then form productive signaling complexes. The postprandial FGF19 (FGF15 in mice) is produced in ileum under the regulation of nuclear farnesoid X receptor (FXR), and thought to act in liver hepatocytes where FGFR4-KLB resides to negatively regulate bile acids synthesis [8], [21], [24]. Vitamin D and the vitamin D receptor regulate the expression of FGF23 in bone that in turn negatively regulates minerals metabolism in the kidney that expresses FGFR1 and KL [10].

As the FGF19, FGF21 has been reported to regulate glucose and lipid metabolism and energy balance [25], [26]. Treatment with FGF21 corrects metabolic disorders such as hyperglycemia, hyperlipidemia and insulin resistance in rodent and primate models of diabetes and obesity [27], [28], [29], [30]. However, the predominant endocrine axis in which FGF21 is produced in one tissue and then targets the other tissues underlying these beneficial effects is not clearly understood. It is reported that FGF21 is induced preferentially in the liver under the control of PPARα in response to fasting, ketogenic diet and in type 2 diabetes and obesity [31], [32], [33], [34], [35]. It is also expressed in some extra-hepatic metabolic and endocrine tissues. In white adipose tissues and cultured adipocytes, FGF21 expression is upregulated by PPARγ agonist or under conditions including fasting and high fat diet feeding [36], [37], [38]. Skeletal muscle or cultured muscle cells were reported to express and secrete FGF21 under insulin stimulation and dependent on AKT1 pathway [39], [40]. Thermogenic activation can induce brown adipose tissue to express FGF21 under ATF2 control [41]. Adult thymus may also expresses FGF21 [42]. On the other hand, several tissues have been implied as the potential targets of FGF21 action. Most reports suggested that in adipocytes and white fat, FGF21 stimulates glucose uptake via upregulation of GLUT1 expression [28], [36], but were discrepant on whether it stimulates or inhibits lipolysis [28], [43], [44], [45]. Several reports in FGF21 transgenic and knockdown mice demonstrated that FGF21 may play a role in the physiological response of the liver to fasting and ketogenic diets through stimulating ketogenesis and triglycerides clearance in the liver [33], [46], [47], but other studies such as with FGF21 ablation did not confirm the observations in the liver and suggested that FGF21 primarily stimulates lipolysis during fed state but inhibits upon fasting in white fat [44]. The effects of FGF21 in the liver are assumed through an autocrine or paracrine action that controls ketogenesis [31], [33], [48], glycemia via regulating glucose flux and insulin sensitivity [27] and gluconeogenesis through induction of PGC-1alpha expression during prolonged fast [47]. FGF21 may also improve pancreatic β-cells function and survival [49], stimulate skeletal muscle glucose uptake [50], stimulate thermogenesis of brown fat by increasing the expression of thermogenic genes and enhancing the total and uncoupled respiration and glucose oxidation [32], and stimulate the hypothalamus or central nervous system for energy expenditure and reduction of obesity in rat DIO model [51].

FGFR1 and FGFR4 are two major receptor isotypes implicated by the majority of reports in the action of endocrine FGF21 and FGF19. FGFR1 is predominantly expressed in adipose tissue and adipocytes, and FGFR4 is predominantly in the liver and hepatocytes, all of which co-express the KLB [17], [21], [23], [52]. As measured by crude affinity pull-down of ectodomains of FGFRs or by ERK1/2 activation in vitro, both FGF19 and FGF21 could bind and activate FGFR1 in the presence of KLB [9], [17], [23], [53], [54]. Unlike FGF19, although FGF21 could also bind to FGFR4-KLB weakly, it was unable to activate FGFR4-KLB and to stimulate the liver response [17], [23], [44]. Even though FGF21 is expressed predominantly in the liver, it could activate the response of fat tissue or adipocytes but not the liver [17], [44]. These results differ from several reports that suggested the stimulatory activity of FGF21 for the liver response [27], [31], [33], [47], [48]. To better understand the receptor and tissue specificities of FGF21, which are the core constituents of its endocrine metabolic pathway, in comparison to FGF19 and the canonic FGF1, we employed in this study several approaches including the sensitive radio-labeling and quantitative cell surface receptor competition binding and activation, receptor downstream signaling relay and the expression of the early response gene. We found that in presence of KLB, FGF19 binds and activates both FGFR1 and FGFR4 to a comparable extent, while FGF21 binds and activates the FGFR1 that resides in adipose tissues but comparably not the liver FGFR4. Our results support the idea that among endocrine and metabolic tissues, hepatic FGF21 directly and preferentially acts on fatty tissues but less likely or less significantly on the liver.

## Results

### FGF21 interacts only with FGFR1 with high affinity in presence of KLB while FGF19 with both FGFR1 and FGFR4

**Figure 1:**
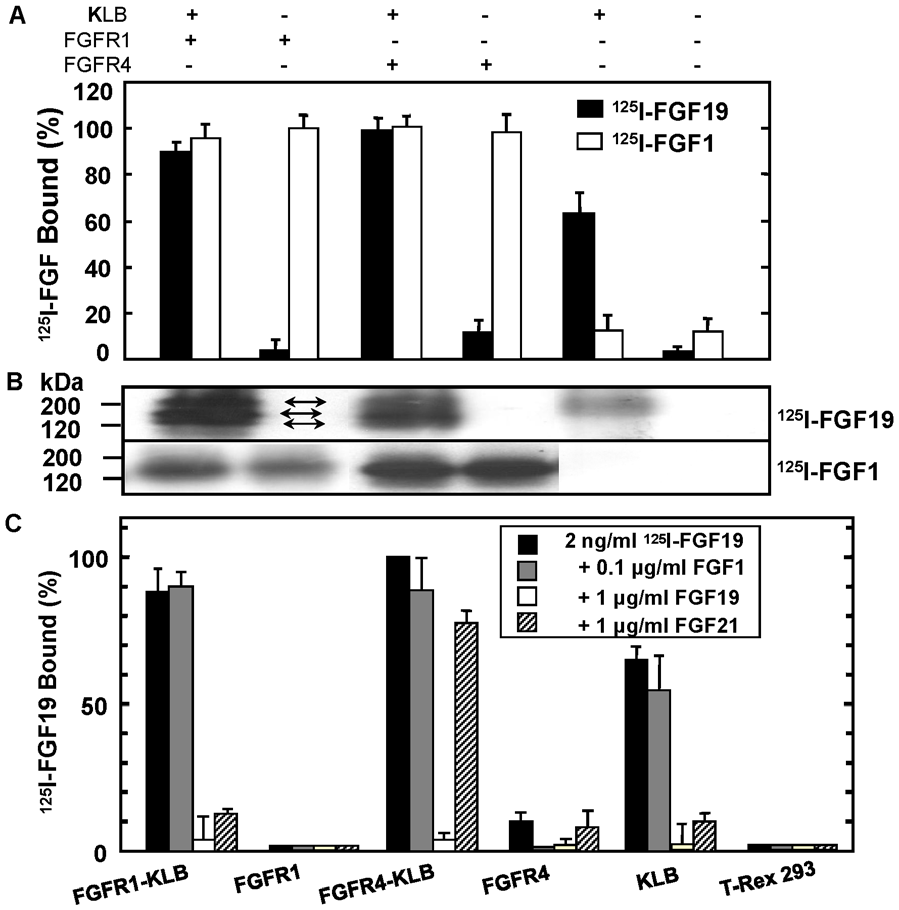
Receptor specificity of FGF1, FGF19 and FGF21. (A) Comparative binding properties of FGF19 and FGF1. Parental T-Rex 293 cells and cells expressing FGFR1-KLB, FGFR1, FGFR4-KLB, FGFR4 and KLB upon 30 ng/ml Tet induction for FGFRs were incubated with 2 ng/ml ^125^I-labeled FGF1 (white rectangle) or ^125^I-FGF19 (black rectangle) as indicated. Cell surface bound radioactivity was determined by γ–counter. Data are shown as mean ± s.d. of three independent experiments. (B) Covalent Affinity chemical cross-linking was performed as described [4]. Cross-linking bands were indicated by bidirectional arrows. (C) Receptor binding properties of FGF21 revealed by competition binding with ^125^I-FGF19. FGF21, FGF19 and FGF1 at 1 µg/ml were incubated in the presence of 2 ng/ml ^125^I-FGF19 with parental cells and cells expressing FGFR1-KLB, FGFR1, FGFR4-KLB, FGFR4 and KLB. Specific cell surface binding and statistic analyses were determined as above.

**Figure 2:**
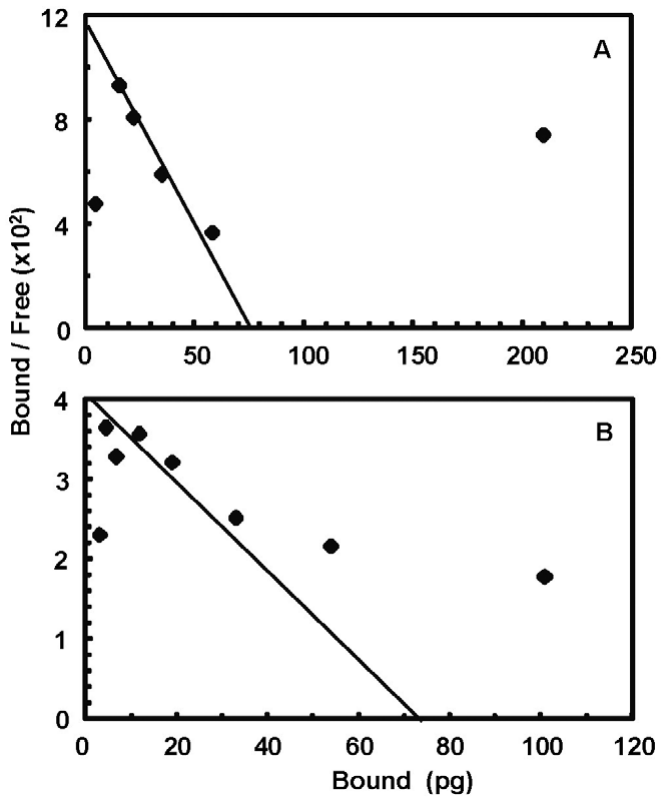
Analyses of binding kinetic and affinity constant. T-Rex 293 cells expressing inducible FGFR1 and constitutive KLB (A) and hepatoma cells HR4 expressing FGFR4 and reduced level of KLB (B) were maintained in DMEM medium with 5% FBS. Binding kinetic analysis of radiolabeled FGF19 to the FGFR-KLB complexes on cell surfaces were done as described in the Materials and Methods. The binding affinity constant values were determined by the Scatchard plot.

To gain insights into the receptor and tissue specificity of FGF21 action as compared to FGF19 and canonic FGF1, we first performed *in vitro* sensitive and quantitative receptor binding kinetic analyses. It has been known the direct contact interaction between transmembrane FGFR1/4 and KLB by mutual affinity pull-down and identification by nano-LC MS/MS [4], [28]. We found that FGF19 bound equally well to FGFR1-KLB and FGFR4-KLB, but not to FGFR1 alone although there is 10% binding to FGFR4 alone consistent with previous report (Figure 1A) [4]. Covalent affinity cross-linking revealed three cross-linking bands resulted from ^125^I-FGF19 bound to FGFR1 and KLB co-expressing cells (Figure 1B, bidirectional arrows), similar to what we observed in the interaction of ^125^I-FGF19 with FGFR4 and KLB, but not with FGFR1 or FGFR4 alone [4]. The successful covalent affinity crosslinking also indicated that our recombinant FGF19 was sufficiently active for interaction with FGFR-KLB (see also Figure 2 described below), similar to FGF1 with or without KLB.

Since iodination with the same procedure appeared to inactivate FGF21, we used the labeled FGF19 as the binding tracer and unlabeled FGF21 as a competitor to probe the receptor binding activity of FGF21. 2 ng/ml of iodinated FGF19 could make equally detectable binding to that of 2 ng/ml iodinated FGF1. 1 µg/ml FGF21 competes with ^125^I-FGF19 for binding to FGFR1-KLB or KLB alone with over 90% efficiency, indicating both recombinant FGF19 and FGF21 had comparable activity towards FGFR1-KLB and KLB. However, at this high concentration there is only 10–20% efficiency for FGF21 to bind FGFR4-KLB. No competition binding can be detected to FGFR4 alone to which 10% of ^125^I-FGF19 bound as compared to FGFR4-KLB (Figure 1C, bidirectional arrows) [4]. In contrast, although 1 µg/ml unlabeled FGF19 competes with 2 ng/ml ^125^I-FGF19 completely as predicted, FGF1 had no displacement for ^125^I-FGF19 bound to the FGFR1-KLB, FGFR4-KLB, or KLB alone at 100 ng/ml (Figure 1C) or 1 µg/ml (not shown). The weakly bound FGF19 to FGFR4 alone can be displaced by 100 ng/ml FGF1 equally well as FGF19 itself but not FGF21. These results are consistent with the previous report that, ^125^I-labeled FGF1 binds equally well to the surface of cells where FGFR4 are present independent of KLB; in contrast, FGF19 binds to cells that express KLB, which include cells co-expressing FGFR4-KLB and cells expressing KLB alone [4].

It should be noted that the activity of recombinant FGF19 and FGF21 is comparable to those currently used in other groups (personal communication). Radio-iodinated FGF19 exhibited an affinity with a Kd value of 303 pM to the FGFR1-KLB complex expressed on the T-Rex 293 cells [4] (Fig. 2A), and 778 pM to a hepatocellular carcinoma cells expressing FGFR4 and KLB [4], [8] (Fig. 2B). The binding affinity constants of FGF19 are 3–7 times less than those of FGF1 [55], but are better than those from other experiments [53], [54], [56]. Since a same iodination procedure inactivated both mouse and human FGF21, we employed mutual comparative competition binding assay and iodinated FGF19 to address their receptor binding ability and specificity; however, it would be desirable in the next step to overcome this iodination problem, so the native binding constant can be deduced and compared to FGF19 in the direct binding assay on cells expressing endogenous FGFR and KLB.

### Dose-dependent competition of FGF21 with FGF19 for binding to FGFR1-KLB but comparably not to FGFR4-KLB

**Figure 3:**
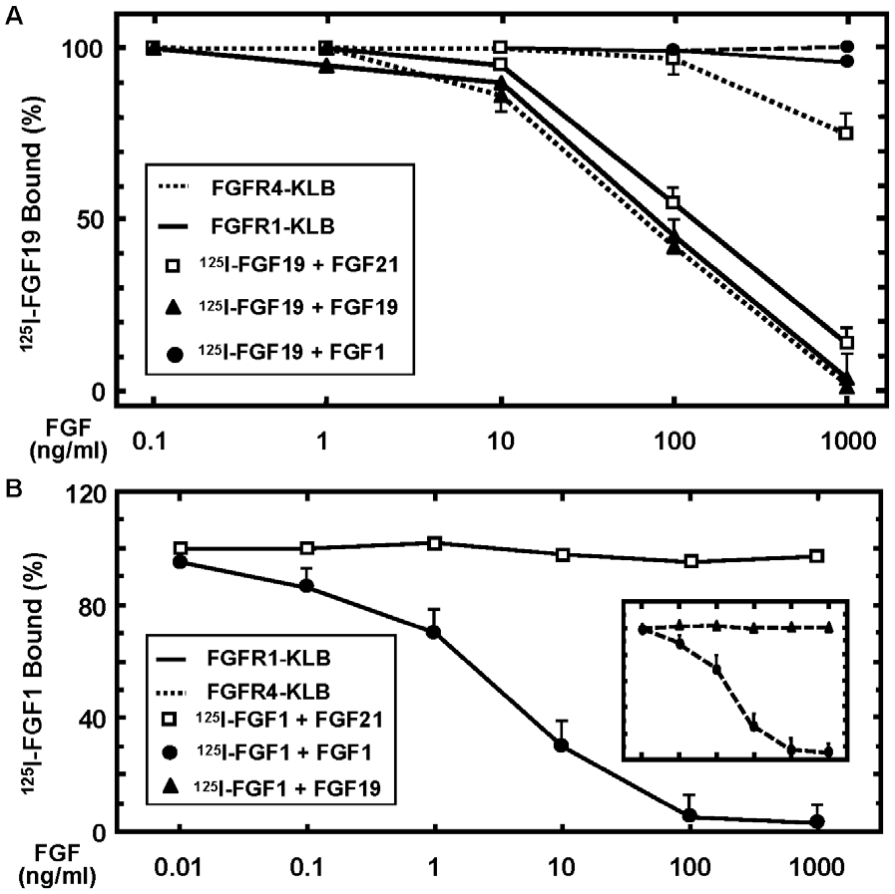
Dose-dependent differential binding of FGF21, FGF19 and FGF1 to FGFR1-KLB and FGFR4-KLB. (A) Competition ability of FGF21 and FGF1 with FGF19 to bind FGFR-KLB. Graded concentrations as indicated for FGF21 (open square), FGF19 (filled triangle) and FGF1 (filled circle) were added together with 2 ng/ml ^125^I-FGF19 to the cells expressing FGFR1-KLB (solid line) or FGFR4-KLB (dot line) after 30 ng/ml Tet induction overnight, the remaining specific bindings of ^125^I-FGF19 were determined as described in Figure 1A. (B) Competition ability of FGF21 with FGF1 to bind FGFR1-KLB. Graded concentrations as indicated for FGF21 (open square), FGF19 (not shown) and FGF1 (filled circle) were added together with 2 ng/ml ^125^I-FGF1 to the cells expressing FGFR1-KLB (solid line) or FGFR4-KLB (Inset), the remaining specific bindings of ^125^I-FGF1 under these conditions were determined as described above.

To determine quantitatively the interaction nature of FGF19 and FGF21 with FGFR1 and FGFR4, we assessed the dose-dependent competition of FGF21 with FGF19 for receptor binding. ^125^I-FGF19 was co-incubated with graded concentrations of FGF21 on cells co-expressing KLB and inducible FGFR1 or FGFR4, respectively [4]. The remaining binding of FGF19 was measured quantitatively by radio-activity. FGF21 couldn't compete with ^125^I-FGF19 for FGFR4-KLB interaction, with a half-maximum effective concentration that is out of valid calculation range. In marked contrast, FGF21 competes for binding to FGFR1-KLB with a half-maximum concentration of about 150 ng/ml, which is comparable to 110 ng/ml for unlabeled FGF19 for binding to both FGFR1-KLB and FGFR4-KLB (Figure 3A).

On the other hand, FGF1 at all concentrations tested couldn't compete with ^125^I-FGF19 for binding to FGFR1-KLB and FGFR4-KLB (Figure 3A). Conversely, both FGF21 and FGF19 couldn't compete with ^125^I-FGF1 for binding to either FGFR1-KLB (Figure 3B) or FGFR4-KLB (Figure 3B inset) at all concentrations tested. This strongly indicates that the biochemical mechanisms underlying the receptor interaction are likely different between the heparin-binding canonic FGFs and KLB-binding endocrine FGFs.

### Interaction of FGF19 and FGF21 with KLB

**Figure 4:**
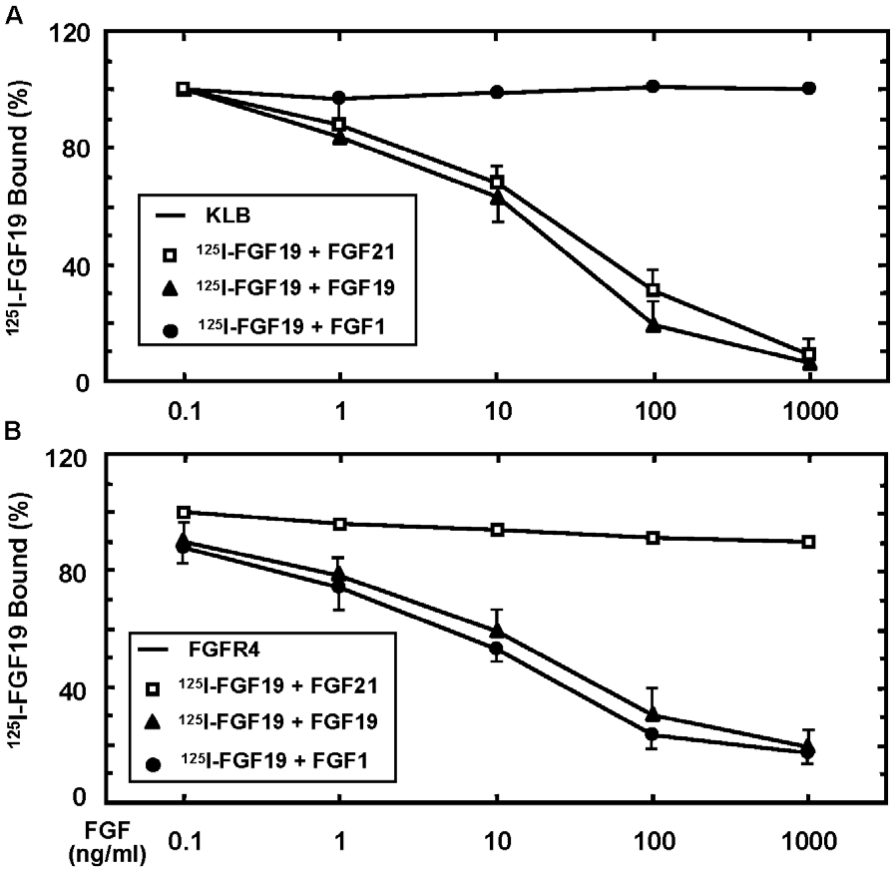
Dose-dependent differential binding of FGF21, FGF19 and FGF1 to KLB or FGFR4 alone. (A) Interaction of FGF21, FGF19 and FGF1 with KLB. Graded concentrations as indicated for FGF21 (open square), FGF19 (not shown) and FGF1 (filled circle) were added together with 2 ng/ml ^125^I-FGF19 to the cells expressing KLB alone, and the remaining specific bindings of ^125^I-FGF1 under these conditions were then determined. (B) Competitive binding of FGF21 and FGF1 with FGF19 to FGFR4 alone. Graded concentrations as indicated for FGF21 (open square), FGF19 (not shown) and FGF1 (filled circle) were added together with 2 ng/ml ^125^I-FGF19 to the cells expressing FGFR4 alone, and the remaining specific bindings of ^125^I-FGF1 under these conditions were then determined.

One notable feature of FGF19 is its high-affinity binding to KLB alone but not FGFR alone [4] (Figure 1A). To further assess whether FGF21 has the same ability to bind KLB as FGF19, graded concentrations of FGF21 were used to compete with ^125^I-FGF19 binding to KLB. FGF21 was able to displace ^125^I-FGF19 from KLB in a dose-dependent manner that is similar to the unlabeled FGF19; in contrast, FGF1 was unable to do so at all concentrations determined (Figure 4A). These results are consistent with the role of the C-terminus of FGF19 and FGF21 in the interaction with KLB [53], [54], [57], and suggest a mechanism that endocrine FGFs form complex with FGFRs through in part the binding to KLB with high affinity but not to HS motifs, while canonic FGFs need only HS motifs for the complex formation with FGFRs.

Since our results shown that FGF19 could bind to FGFR4 alone with 10% efficiency but not FGFR1, we ask whether such a weak binding to FGFR4 is related to the mechanism of FGF1 binding. In fact, FGF1 displaced ^125^I-FGF19 from FGFR4 completely in a dose dependent manner similar to that of unlabeled FGF19, while FGF21 was unable to do so (Figure 4B). This is in contrast to the inability of both FGF19 and FGF21 to displace ^125^I-FGF1 from binding to FGFR4, FGFR4-KLB or FGFR1-KLB (Figure 3B). These data indicates that there may be a fundamental difference between the canonic FGF1 and endocrine FGF19 or FGF21 in the interaction with FGFRs, and such a weak FGF19 interaction may contribute to the receptor selectivity. As the interaction of both FGF19 and FGF21 with the binary FGFR-KLB complex is stronger than with KLB, and the N-terminus of FGF21 is important for interaction with FGFRs measured by the downstream ERK1/2 activation but not without the presence of KLB [53], [54], our results suggest that the integration of KLB is the key mechanism in the productive complex formation for the endocrine FGF19 and FGF21 with the canonic HS-FGFRs pre-complex residing across the plasma membrane.

### FGF21 activates downstream signaling of FGFR1-KLB but not FGFR4-KLB

**Figure 5:**
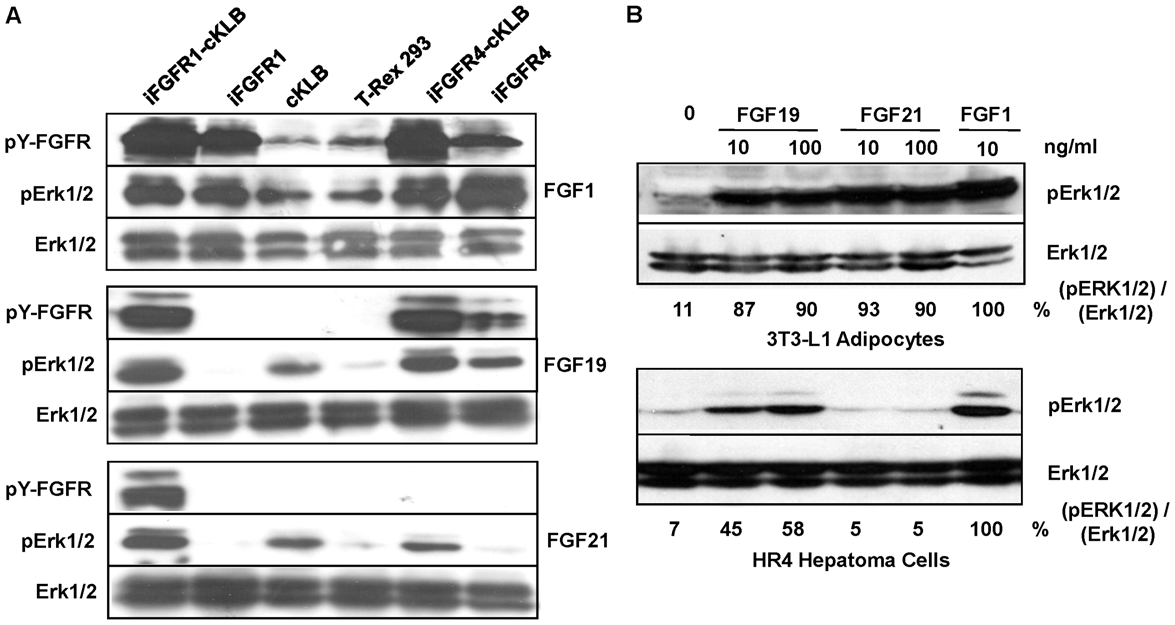
Differential activation of FGFRs and downstream MAPKs by FGF19 and FGF21. (A) Tyrosine phosphorylation of FGFR and activation of ERK1/2 in engineered T-Rex 293 cells [4]. Expression of FGFR1 and FGFR4 was induced with 30 ng/ml Tet overnight. Cells were stimulated with 100 ng/ml FGF21, FGF19 and FGF1 in presence of 1 µg/ml heparin, cell lysates in 1×SDS sample buffer were used for immunoblot analyses using antibodies as indicated. The identity of FGFR1 and FGFR4 was pre-determined by their respective antibodies (not showed). (B) Responses of adipocytes and hepatocyte-like cells to the stimulation of FGF19 and FGF21. Mature 3T3-L1 adipocytes and HR4 hepatoma cells after overnight serum-starvation were treated by FGF21, FGF19 and FGF1 at the concentrations as indicated, and cell lysates were used for immunoblot analyses for MAPK activation as described above. The average relative activation level of Erk1/2 for each cell type under different stimulation condition is expressed as the percentage to the peak activation of Erk1/2 treated with FGF1 after normalized as ratio of pErk1/2 to total Erk1/2.

To determine whether the detectable surface receptor interaction leads to receptor downstream signal transduction, we assessed the downstream ERK1/2 activation in the same T-Rex 293 cells on which we performed the binding analyses. The ectopic expressions of FGFR1 and FGFR4 were induced by 10–30 ng/ml Tet overnight to avoid extensive apoptotic effect in KLB co-expressing cells [4]. Consistent with the binding profile, FGF1 activates FGFR1-KLB, FGFR1, FGFR4-KLB and FGFR4 equally well but not KLB, and KLB has no inhibitory or stimulatory effects on FGF1 binding (Figure 1 and 3) and activity (Figure 5A). FGF19 activates both FGFR1-KLB and FGFR4-KLB efficiently and FGFR4 alone weakly but not FGFR1 alone as revealed by FGFR autophosphorylation and pERK1/2 (Figure 5A); while in marked contrast, although FGF21 bound to FGFR4-KLB very weakly, it could only activate the FGFR1-KLB expressing cells (Figure 5A), not the FGFR4-KLB, FGFR4 or FGFR1 expressing cells over the response level of the cells expressing KLB alone. Both FGF19 and FGF21 activate the KLB-expressing cells, albeit with a weaker potency, which is consistent with other reports [17], [21] as the T-293 cells expressed the endogenous FGFR1 at a low level (unpublished observation) [17]. These results further support the idea that FGF21 is likely unable to activate FGFR4-KLB expressing cells.

To investigate whether the effective receptor binding in transfected T-293 cells represents biological or physiological interactions, and therefore, activities, we determined the biological responses of cells expressing the endogenous FGFR and KLB to FGF19 and FGF21 stimulation with a comparable concentration used in the aforementioned binding assays. In differentiated 3T3-L1 adipocytes that co-express predominantly the FGFR1-KLB, FGF19, FGF21 and FGF1 activate the ERK1/2 to an equal extent, while in hepatoma cells co-expressing preferentially the FGFR4-KLB but not FGFR1-KLB, FGF21 is unable to activate the ERK1/2 response as compared to FGF19 and FGF1 (Figure 5B). This again indicates a potential difference in the endocrine activities of FGF19 and FGF21. In the *in vivo* situation, FGF19 may activate a broad type of tissues other than the liver, while FGF21 may restrict to the tissues related to adipose property or expressing predominantly the FGFR1-KLB.

**Figure 6:**
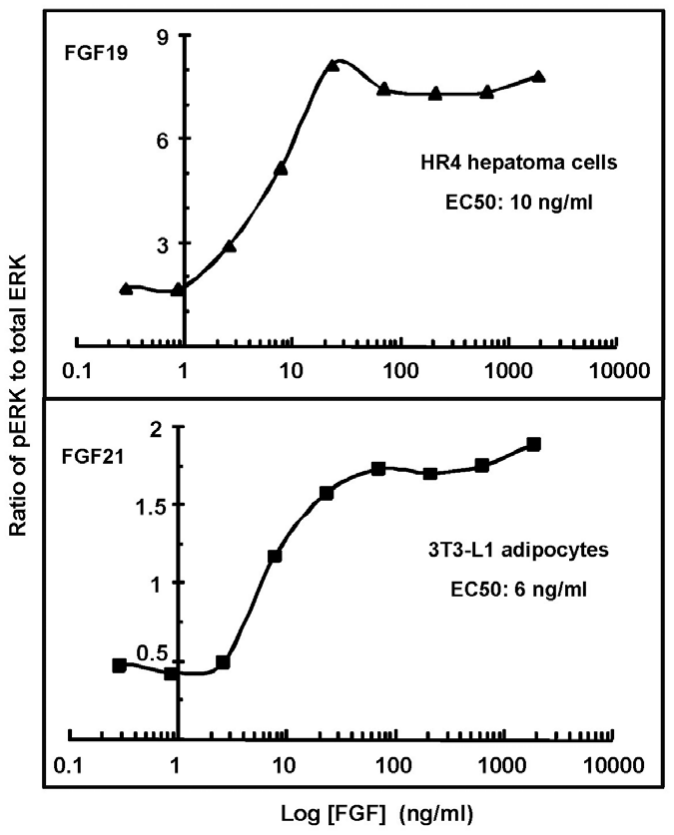
Dose-dependent activity potential of FGF19 and FGF21. Cells expressing endogenous FGFR4-KLB and FGFR1-KLB as indicated were stimulated by different concentrations of FGF19 and FGF21, respectively, for 10 minutes in 37°C culture. Cell lystes were then used to determine the pERK1/2 levels under these conditions by western-blotting. The pErk1/2 level for each cell type under each stimulation condition is expressed as normalized arbitral units of ratio of pErk1/2 to total Erk1/2. The half maximal effective concentration (EC50) is then calculated in the logarithmic plot.

Furthermore, ERK1/2 activation levels in response to graded dosages of FGF19 and FGF21 in these cells indicated the half-maximum concentrations for FGF19 and FGF21 were 10 and 6 ng/ml, which is 0.5 and 0.3 nM, respectively, given that the molecular weight of both is about 20 kDa (Figure 6). These concentrations are comparable to the recombinant FGF21 and FGF19 used in other reports [53], [54], [56].

### FGF21 activates predominantly the response of white adipose tissue

To gain further insights into how FGF19 and FGF21, which differ in specific FGFR-KLB interaction, differentially impact *in vivo* endocrine metabolic effects, we determined their activities in regulating the c-Fos gene expression, which is a marker in early response to FGF19 stimulation [21], in different endocrine and metabolic tissues including the liver and white adipose tissue that are the two major types of tissues being implicated predominantly in their actions. However, current reports are discrepant on whether there is a liver effect of the FGF21 endocrine action. It is also not clear whether there is an direct adipose effect of FGF19, even though it was known that FGF19 exhibited anti-obesigenic and anti-diabetic activities [58], [59].

**Figure 7:**
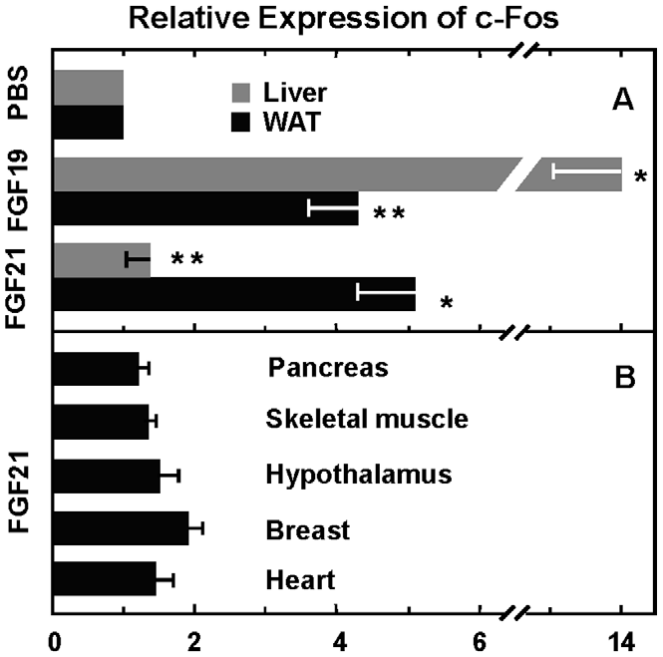
Differential tissue-specific responses to FGF21 and FGF19 as measured by the expression level of early responsive gene c-Fos. Thirty age- and weight-matched mice (2×3×5) were fasted for 24 hrs with water freely available, and then injected intraperitoneally with FGF21, FGF19 (0.5 mg/Kg body weight) and PBS vesicle control as indicated. After 20 min, liver and adipose tissue (A) and several other endocrine and metabolic tissues (B) were isolated and processed for RNA purification. Quantitative PCR was used to assess the expression of c-Fos in response to different treatments (n = 5).

Recombinant FGF21 at 0.5 mg/Kg were injected into mouse intraperitoneally as compared to FGF19 and vehicle PBS, and after 20 min tissues were isolated for qPCR gene expression analyses. FGF21 and FGF19 stimulated c-Fos mRNA expression in mouse liver at 1.3 and 13.8 times over the PBS control, respectively (Figure 7A). The liver response to FGF19 is more than 10 times that to FGF21. In contrast, FGF21 and FGF19 stimulated c-Fos mRNA expression in WAT at 5.1 and 4.3 times over the control, respectively (Figure 7A). The response of WAT to FGF19 is about 80% that to FGF21. The responses of other tissues including pancreas, skeletal and heart muscles and hypothalamus to FGF21 stimulation are relatively very low or undetectable (Figure 7B). Note that the breast, which contains mostly the adipose tissue, is the second highest responder. In all the cases, the response of the liver to FGF21 is the lowest and close to vesicle control. A thorough comparison of differential responses among all aforementioned tissues to FGF21 and FGF19 would further provide insights into their differential biological effects, which will be undertaken in tissues from wild-type, KLB−/−, FGFR1 conditional ablation and FGF21 transgenic mice after crossbreeding into a similar strain background.

**Figure 8:**
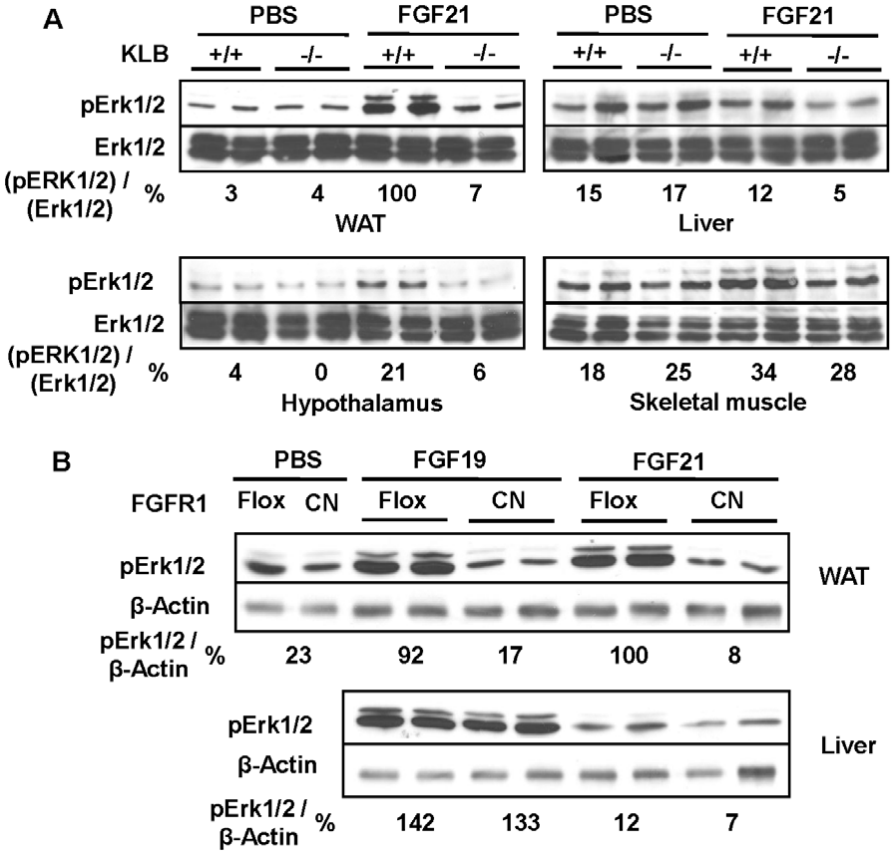
Differential tissue-specific responses to FGF21 and FGF19 as measured by the activation level of Erk1/2. Endocrine FGF treatment and tissue collection in mice were as described in Figure 7. The pErk1/2 levels in response to FGF21 in different tissues from wild-type and KLB−/− mice (A) as compared to PBS control, or in the adipose tissue and liver from the FGFR1 floxed (Flox) and CN mice (B) as compared to FGF19 and PBS were determined by immunoblot analyses. Data are representatives of 4–6 mice for each treatment scheme in each genotype group. The average relative activation level of Erk1/2 for each type of tissue is expressed as percentage to the peak activation in WAT of the Flox mice treated with FGF21, after normalized as ratio of pErk1/2 to total Erk1/2 (A) or ratio of pErk1/2 to β–Actin (B).

**Figure 9:**
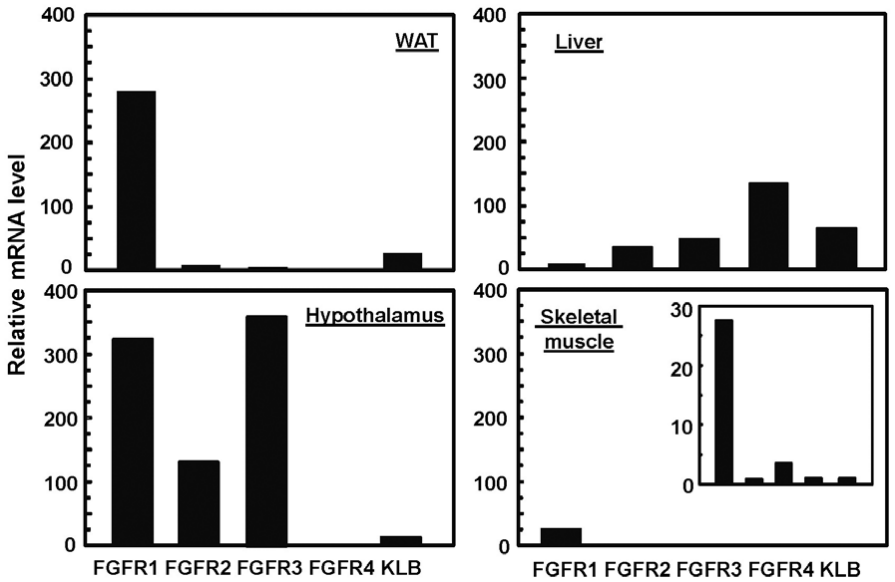
Tissue-specific expression of FGFRs and KLB. Tissues as mentioned in Figure 8 were isolated from healthy C57/BL6, and total RNA extracted was used for qPCR analyses of the expression of FGFR1–4 and KLB. Normalized expression levels were expressed as folds of difference relative to that of β-Actin detected in each sample, and multiplied by 10^4^ for graphic presentation. Data were presented as the mean of triplicates ± S.D. Inset: small scale plot on y-axis of expression levels in skeletal muscle.

Consistent with the acute c-Fos expression response, treatment of mice with FGF21 for 20 minutes stimulates peak activation of Erk1/2 [28] in the WAT (Figure 8A), but not in the liver and only very weakly in hypothalamus and possibly skeletal muscle as well (Figure 8A). These effects are substantially abolished in mice with gene deletion for the cofactor KLB, as indicated by the percent changes of pErk1/2 relative to total Erk1/2 (Figure 8A). Furthermore, the conditional ablation of FGFR1 in adipocytes in WAT by aP2 Cre (manuscript in preparation) also completely abrogates the Erk1/2 activation by both FGF21 and FGF19 (Figure 8B), as compared to the FGFR1 floxed tissue and PBS vehicle control. In the liver unaffected by the adipocyte-specific ablation of FGFR1, Erk1/2 activation in response to FGF19 remains unchanged, while the response to FGF21 is relatively insignificant or undetectable (Figure 8B), similar to the baseline control from PBS as the delivery vehicle (not shown). This response profile is also consistent with the differential tissue specific expression of FGFR and KLB (Figure 9). The expression of FGFR1 is high in WAT and hypothalamus. FGFR4 is only expressed in the Liver, not in the WAT, hypothalamus and skeletal muscle. In contrast, KLB is expressed high in both WAT and liver, but low in the hypothalamus. Skeletal muscle expresses very low level of FGFR1 predominantly as compared to the WAT and hypothalamus. The rather weak response of hypothalamus to exogenous FGF21 stimulation for 20 minutes may be due to the blood-brain barrier and the low level of KLB expression (Figures 8 and 9). The levels of KLB and FGFR1 in the tissues from KLB−/− mice and WAT from FGFR1 CN mice, respectively, are below the detection limit, the arbitrary cycle time of 34 in qPCR analyses. Since FGF21 is preferentially expressed in the liver, our results indicate not only the critical role of KLB and FGFR1 as the biological receptor complex in transducing the signal of FGF21 and FGF19, but also a major emerging endocrine metabolic axis from the liver to adipose tissues.

## Discussion

As a single transmembrane protein, KLB plays an important role in regulating bile acid synthesis, as revealed by genetic ablation in normal condition [18], [60]. KLB−/− mice exhibited elevated biosynthesis and secretion of bile acids through upregulation of Cyp7a1 expression, a rate-limiting enzyme in the bile acid anabolic pathway. This phenotype resembles those of the FGFR4 and FGF15 (FGF19 in human) knockout mice [11], [24]. Mice deficient in KL exhibited a variety of aging-like phenotypes [19], many of which phenocopy those observed in FGF23−/− mice [61]. These studies uncovered the biological connections among the conventional HS-FGFR, the KL family, and the FGF19 subfamily that function in convergence in signal transduction pathways for commitment of metabolic regulation [9], [10], [56], [62] rather than grossly the growth control. Like canonic FGFs, the endocrine FGF19, FGF21 and FGF23 remain to signal through FGFRs but only in the presence of the KL family. The underlying mechanism for the KL family integration and the consequence resulted from is still unclear. In this study, we found that although FGF19 and FGF21 are unable to bind HS-FGFR alone, they are able to bind through KLB with high affinity. The binding of FGF19 and FGF21 to KLB and FGFR-KLB is not affected by the presence of canonic member FGF1; similarly, the binding of FGF1 to FGFR and FGFR-KLB is also completely resistant to the presence of FGF19 and FGF21. The presence of KLB confers even higher affinity for the FGF19 subfamily on the HS-FGFR complex than KLB alone. These data suggest a mode for the endocrine FGF-initiated FGFR signaling complex formation that is different from that initiated by canonic FGFs.

We speculate that the binding sites on FGFR for FGF1 and FGF19/21 may be not all the same, or may require additional composite ones during the complex assemblage [16], [63]. It is also possible that FGF1 could reject the KLB from the complex, or that KLB has no effect on FGF1-FGFR complex formation even when present. The latter possibility appears to be supported by our data that no additional cross-linked complexes were detected with labeled FGF1 in the co-expression cells. This may explain why FGF21/19 cannot compete with FGF1 binding even in presence of KLB. On the other hand, the fact that FGF1 cannot compete with FGF19 may indicate a FGFR-independent binding site of FGF19/21 on KLB. Our results imply that KLB functions as a key regulator of FGF19 and FGF21 not only by promoting their high-affinity binding to and subsequent activation of FGFRs, but also by determining their tissue-specific activity where KLB and FGFRs are specifically co-expressed [17], [21].

The differences in formation of the active canonic and non-canonic complexes may underlie their downstream functional divergence [1], [4], [64], [65]. Further studies should shed light on the mechanisms by which canonic FGFs and endocrine FGFs coordinate local cell proliferation and metabolic function during developmental stages and in pathophysiological circumstances. This may be through regulating the ratio of FGF receptor to the cofactor KLB, therefore, switching the end-effects between the cell proliferation promoted by FGFR free of KLB and the cell metabolism controlled by the integrative FGFR-KLB complex. It is likely that the integration of KLB and endocrine FGFs in specific tissues alters the major downstream signal effectors or pathways, therefore, results in differential end-effects.

Although ileum FGF15/19 plays a primary role in negatively regulating hepatic bile acids synthesis, there is so far no reported adipose tissue phenotype in the FGF15−/− mice. However, FGF19 (FGF15 in mice) administration or overexpression was reported to have a profound impact on adiposity and diabetic parameters, through yet unclear mechanisms in term of tissue and molecular targets [21], [58], [59]. These effects are markedly similar to those of FGF21 administration or overexpression through regulating lipid, glucose and energy metabolism. Both FGF19 and FGF21 reach to target tissues through the endocrine mechanism, the circulation. In particular, FGF19 has been proposed to reach to the liver through portal vein from intestine; therefore, it likely that through circulation, the adipose tissues will be a secondary target of FGF19, even though a significant portion of FGF19 from intestinal producing site may be trapped in the liver through the dominant enterohepatic circulation. It is conceivable that FGF19 may coordinate metabolism in the adipose tissues and liver in response to the prandial stimulation. This is also supported by our current data; however, more in vivo studies are needed to address this important physiological possibility.

FGF21 reportedly has no effect on expression of cyp7a1, a key enzyme in hepatic bile acids synthesis (17), which is a hallmark of FGFR4-KLB function in the liver; however, it plays notable roles in lipid and glucose metabolism and thus is proposed as a novel pharmacotherapy for obesity and diabetes. As of FGF19, the mechanisms underlying the beneficial effects of FGF21 are also unclear. Reports are discrepant on whether FGF21 has roles directly in the liver for regulation of ketogenesis, triglycerides clearance and glucose disposal, and on whether FGF21 stimulates or inhibits lipolysis in white fat [17], [27], [31], [33], [44], [47]. Reports including ours (manuscript in preparation) are consistent on the predominant co-expression of FGFR4-KLB in the liver, hepatocyte or hepatocyte-derived cells, and of FGFR1-KLB in adipose tissues or mature adipocytes [17], [21]. One of the keys to clarify these important issues is to see how different tissues respond at the early stage of FGF21 stimulation. In this study, we showed that although FGF19 interacts with both binary FGFR1-KLB and FGFR4-KLB with high affinity, FGF21 is able to bind only the FGFR1-KLB but not FGFR4-KLB with a high affinity comparable to that of FGF19 binding. This differential molecular interaction underlies the different tissue response profiles for FGF19 and FGF21. FGF21 only effectively activates the responses of the adipose tissue and adipocytes but not the liver where these binary complexes are differentially present [17], [28], [44], [52]; on the other hand, FGF19 activates the responses of both the liver and adipose tissue and the derived cells. These data are consistent with our previous observation that mice with liver specific overexpression of constitutively active FGFR1 gain a suppression of bile acids synthesis that is similar to the constitutively active FGFR4 overexpression [66]. Our results thus support a direct endocrine metabolic role of FGF21 in fatty tissues but less likely in the liver, while FGF19 may have also a role in fatty tissue, beyond the liver as a primary target [59], [67]. The reported effects of FGF21 on the metabolic parameters in the liver are more likely from a secondary indirect response of the liver to the direct metabolic effects of FGF21 on other tissues, in particular the fatty tissues, in a physiological concentration. This is consistent with the central roles of the liver in monitoring, regulating and responding to the ever-changing metabolic states of the whole body and many other individual tissues. The results that FGF21 is primarily expressed in the liver in response to fasting and pathological conditions such as fatty accumulation and the obesity and diabetes [68], [69], and that FGF21 targets primarily the fatty tissues, indicate an emerging endocrine metabolic pathway from the liver to fatty tissues in regulating the lipid, glucose and energy metabolic homeostasis. However, this doesn't exclude a possibility that under certain condition, the weakly expressed FGFR2 and/or FGFR3 beyond the dominant FGFR4 may help the liver to respond to FGF21 in an autocrine/paracrine fashion although likely at a much reduced level. Other tissues, such as the breast, hypothalamus, pancreas and muscle may also respond to FGF19/21 either directly or indirectly under certain physiological and altered pathological states. Whether FGF21 has a possible KLB-independent role *in vivo* or whether FGF21 stimulates the responses of other tissues where KLB expression is low or undetectable, as some studies have suggested for FGF19, is now an open question.

Interestingly, although KLB−/− mice exhibited bile acids phenotype resembling that of FGFR4−/− and FGF15−/−, these mice have not been reported under normal diet condition a metabolic abnormality phenocopying that of FGF21−/−. This does not exclude such a possibility under other pathological or diet stress conditions. The combination of tissue expression specificity and molecular receptor-cofactor interaction specificity determine the eventual physiological outcome. KLB conceivably has functions that overlap with as well as diverge from FGF21. These functional difference and similarity will be balanced to present an overt phenotype under different physiological and pathological states. Tissue specific ablation approaches should be very useful to dissect these difference and similarity issues.

We observed that the binding modes of the canonic FGF1 and endocrine FGF19 and FGF21 to FGFRs likely possess similar as well as different elements. The binding of FGF1 to FGFR-KLB appears to be not affected by the presence of FGF19 or FGF21. Furthermore, FGF1 binds FGFR and FGFR-KLB directly in the presence of HS motifs with high affinity, while FGF19 and FGF21 cannot bind directly to the HS-FGFR but in the presence of the transmembrane KLB. It has been shown that the N- and C-terminuses of FGF19 and FGF21 are required for the interaction with FGFR and KLB, respectively; however, it is obvious that the N-terminus alone is not sufficient for direct interaction with FGFR without the presence of KLB [53], [54], [57]. These imply that the high affinity binding of FGF19 and FGF21 to KLB, which has been shown in a binary complex with FGFR that is ready to be activated by the binding event [4], is likely the first and key promoting step for the subsequent productive complex formation through more interactions with FGFR that produce further higher affinity and more stability. The integration of KLB into the HS-FGFR for transducing the FGF19 and FGF21 stimulation across the plasma membrane is therefore a hallmark event for their metabolic effects. This integration was visualized by the presence of several possible cross-linking bands. It would be interesting to know how the divergence and specificity in the intracellular phosphorylation and selection of signaling relay adaptors are resulted from the extracellular integration.

## Materials and Methods

### Ethics Statement

All mice were housed in the Program of Animal Resources in the Institute of Biosciences and Technology, and were handled in accordance with the *principles and procedure of the Guide for the Care and Use of Laboratory Animals*. All experimental procedures were approved by the Institute of Biosciences and Technology Institutional Animal Care and Use Committee (IBT IACUC) with a protocol #10022 entitled “BetaKlotho-FGFR in the liver”.

### Expression plasmid constructs

Human full-length FGFR1βIIIC (NM_023105) and FGFR4 (AAM13666) in the Tet-on pcDNA4/TO mammalian inducible expression vector, and murine KLB (NM_031180) in pEF1a (a gift from Dr. Kuro-O M) [9] were described previously [4]. Human FGF19 and murine FGF21 in pET28 with 6×His tag at the N-terminus were expressed in BL21 DE3 E. coli, and refolded from inclusion body or directly purified from soluble fraction on Ni-chelating Sepharose chromatography using AKTApurifier (GE HealthCare, MA). Recombinant human FGF21 with a N-terminal 6×His tag with an activity equal to our preparation of murine FGF21 as determined in our ERK activation assay (Figure 6) was from Dr. Xiaokun Li (Wenzhou Medical College, China).

### Cell lines

The establishment, maintaining and protein expression induction of the tetracycline (Tet)-inducible T-Rex-293 cell lines (Invitrogen, CA) expressing the iFGFR1-cKLB, iFGFR4-cKLB, iFGFR1, iFGFR4 or cKLB were described previously [4]. The generation and maintaining of the HR4 hepatoma cell line was also described in our previous study with FGFR4−/− and DEN hepatoma model [4], [70].

### Mouse tissues

The KLB−/− mouse line with an inactivating insertion in the first intron was obtained by gene-trap technology (TIGM, Texas). The homozygous C57/BL6 mice exhibited an increase in hepatic bile acids and about 35% less survival rate than expected, similar to that previously reported [60]. Mice deficient in FGFR1 in specifically the adipose tissue (AdiFGFR1) were generated by using FGFR1f/f (Flox) and aP2-Cre mice [71], [72] (manuscript in preparation). The aP2 promoter is relatively adipocyte-specific. AdiFGFR1 conditional null (CN) mice were obtained from FGFR1f/f mice and aP2-Cre+/w mice, and then breeding with C57/BL6 for more than five generations. qPCR results indicate that FGFR1 was reduced to nearly an undetectable level in adipocytes in WAT.

The male C57/BL6 mice at 5 or 6 weeks old were provided standard feeding and water *ad libitum* according to the protocols approved by the Institutional Animal Care and Use Committee. Before injection mice were fasted overnight by withdrawal of food with water still available. Pure recombinant FGF19 or FGF21 at 0.5 mg/Kg or vehicle (1× PBS) were then injected intraperitoneally into six mice in each group. After 20 min, mice were sacrificed, and the left lobes of the livers, gonadal adipose tissues, hypothalamus, pancreas, skeletal muscle, etc., were isolate, and frozen at −80°C.

### Quantitative PCR analyses

Total RNA was isolated from cells or tissues using Ultraspec RNA Isolation reagents (Biotecx Laboratories, Houston, TX). Equal amounts of total RNA from five mice were pooled and then 5 ug was subjected to the reverse transcription. Primers 5′-ACTCCTTCTCCAGCATGGGCTC and 5′-AGTTGAATCTGTCTCCGCTTGGAG were used to quantify the expression level of c-Fos. Primers pairs 5′-CTGAAGGAGGGTCATCGAAT and 5′-GTCCAGGTCTTCCACCAACT, 5′-CACCACGGACAAAGAGATTG and 5′-TGTCAACCATGCAGAGTGAA, 5′-AGATGCTGAAAGATGATGCG and 5′-ATGATGTTCTTGTGCTTGCC, and 5′-CAGAGGCCTTTGGTATGGAT and 5′-AGGTCTGCCAAATCCTTGTC were for mouse FGFR1, FGFR2, FGFR3 and FGFR4, respectively. Primers 5′-CAGAGAAGGAGGAGGTGAGG and 5′-CAGCACCTGCCTTAAGTTGA were for mouse KLB. These primer pairs were designed by the real-time PCR (TagMan) primer design program from GenScript, all with the Tm of 59°C and the amplicon length of 80–150 bp. Relative gene expression was measured by real-time PCR with 40 cycles using the SYBR Green JumpStart Taq Ready Mix (Sigma) on the Stratagene Mx3000P qPCR system. All measurements were done in triplicate, and relative amounts of mRNA were calculated by the comparative threshold (Ct) cycle method using β-actin as the internal control, and presented as mean±S.E.M using two-tailed unpaired Student's t-test. For the comparison of expression levels of FGFRs and KLB across all different tissues examined, the threshold cycle times were subtracted with 34, which was determined as the cycle limit for detection. Normalized expression levels were expressed as arbitrary folds of difference by dividing by that of β-Actin detected in each sample, and multiplied by 10^4^ for graphic presentation. Data were presented as the mean of triplicates ± S.D.

### Immunoblot analyses

Cells after treatment were lysed in 1× SDS sample buffer. Alternatively, cells were lysed in modified cold RIPA buffer of 20 mM Tris-HCl, pH 7.2, 50 mM NaCl, 1% NP-40, 0.1% sodium deoxycholate, 2 mM sodium orthovanadate (pre-oxidized by H_2_O_2_ prior to use) and 2 mM NaF, and before use, 1 pill each of protease inhibitors and phosphatase inhibitors (Roche, IN) per 10 ml were added. Supernatants after centrifugation were used to perform immunoprecipitation with antibodies and protein A/G agarose. Whole cell lysate supernatants or immunoprecipitates were separated by 10% SDS-PAGE and transferred onto nitrocellulose membranes. The membrane was probed with the antibodies against FGFR4, KLB, ERK and phospho-ERK1/2 (Santa Cruz Biotechnology, CA), and pTyr (Cell Signaling Technology, Inc). For sequential blotting, the wet membranes were stripped by using a solution of 40% methanol and 2% acetic acid for 30 min.

### Cell surface receptor binding, cross-linking and binding constant determination

^125^Iodine labeling of FGF1 and subsequent heparin-sepharose purification were as described [73]. Active recombinant His-tagged FGF19 after iodination was purified by Ni-Chelating sepharose chromatography following the established procedure [4]. Binding was done under specific conditions designed to support specific binding [73] on the surface of T-Rex 293 cells expressing different combinations of FGFR1, FGFR4 and KLB. Specific binding of ^125^I-FGF to the FGFR ectodomain was distinguished and extracted from non-specific binding, and confirmed by covalent affinity crosslinking as described [4], [74]. Scatchard binding kinetic analysis was done in a range of 0.0625 to 8 ng/ml ^125^I-FGF19 on the FGFR-KLB complexes expressed on cell surfaces at 4°C for 30 min. Non-specific and matrix binding was less than 10% of total determined in the presence of 250 µg/ml heparin and 30-fold unlabeled FGF. Affinity constant Kd was estimated by linear least square regression analysis [55], [73]. Experiments were repeated at least three times with samples from at least three independent preparations.

### Reproducibility and statistical analysis

Unless otherwise indicated, each experiment was reproduced at least three times independently with triplicates within each experiment. A representative of three or more experiments is shown in micrographs. Where indicated, the mean and standard deviation (S.E.M) were determined by student t-test.
